# Improving Access to Laparoscopy in Low-Resource Settings

**DOI:** 10.5334/aogh.2573

**Published:** 2019-08-19

**Authors:** Alan J. Rosenbaum, Rebecca G. Maine

**Affiliations:** 1 Department of Obstetrics and Gynecology, University of North Carolina at Chapel Hill, US; 2 Department of Surgery, University of North Carolina at Chapel Hill, US

## Abstract

Laparoscopy has numerous clinical benefits compared to laparotomy. However, a functional laparoscopy program requires significant investment and, as a result, remains unavailable for the majority of the world’s population in low- and middle-income countries. The effort to bring laparoscopy to low-resource settings has produced variable outcomes resulting from the challenges inherent to a complex surgical program. This paper highlights these shortcomings and identifies opportunities to improve future laparoscopy programs.

## Introduction

Since its introduction, laparoscopy has evolved into an indispensable surgical tool. The clinical benefits are well-documented and include reduced blood loss, lower infection rates, shorter hospital stay, faster return to normal activity, improved cosmesis, and less pain and medication use compared to laparotomy [1,2,3,4,5,6,7,8,9]. However, laparoscopy programs are complex and require significant initial and sustained investment including high levels of human capital, technical equipment, and costs [1,7,8,9,10,11,12,13]. Consequently, while laparoscopy has flourished in high-income countries (HICs), it remains unavailable for the majority of the world’s population who live in low- and middle-income countries (LMICs). This article seeks to explore key considerations for stakeholders to develop sustainable laparoscopy programs, including the value of programs in LMICs, optimizing the performance of such programs, and how to best assess the programs.

## Why Does Laparoscopy Matter for LMICs?

Global health investors have historically prioritized infectious disease and maternal-child health over surgical care, despite the fact that nearly one third of the global burden of disease arises from surgical conditions [14,15,16]. The mortality and morbidity benefits of laparoscopy are potentially greater in locations such as LMICs that may lack clean water, sanitation, blood banks, advanced diagnostic imaging, or interventional radiological procedural services [1,5,9,17,18,19,20,21]. In LMICs, laparoscopy has been associated with a greater than 50% reduction in post-operative wound infection rate, hospital stay may be several days shorter, and convalescence can be several weeks faster compared to open surgery [1,7,21,22,23]. This represents meaningful impact on health outcomes, return to economic activity, and hospital and healthcare utilization.

## Current Laparoscopy Program Shortcomings

Laparoscopy programs in LMICs face numerous limitations including a lack of skilled providers; increased operating time; limited resources, equipment, and maintenance capacity; and absence of safe procedure guidelines [6,7,9,11,12,13,21,24,25]. To date, the primary model to develop a laparoscopy program in a LMIC is via partnering with a surgical program in a HIC, where the HIC institution facilitates personnel training, equipment procurement, and clinical guideline development [1,2,8,9,10,21,25].

This model fails to address the aforementioned shortcomings, resulting in foreseeable challenges. Programs offering intermittent in-country clinical support may result in inadequate training and program discontinuation after training concludes [1,21,24,25]. General or gynecologic surgery teams implementing programs in isolation create resource inefficiencies and reduced economies of scale [1,4,9,24]. Reliance on donated devices leads to program interruption when these resources are exhausted [1,6,8,10,24].

## Defining Access

One of the challenges in developing sustainable laparoscopy programs is the limited ability to assess development due to lack of evaluation metrics. To determine a laparoscopy program’s success, it is necessary to obtain the data describing whether a population has adequate access to laparoscopy.

The Lancet Commission on Global Surgery 2030 defined surgical access as safe, affordable, and timely surgical capacity, using three Bellwether procedures (cesarean delivery, laparotomy, and treatment of open fracture) for the metric [14]. Similarly, Bellwether procedures should be used to measure laparoscopy access and data about procedure safety, quality, and costs must be collected. Consistently defining access improves analyses and helps identify deficiencies. Access should be defined as the availability of these procedures, routine and emergent, at all times. We propose diagnostic laparoscopy, appendectomy, cholecystectomy, and gynecologic adnexal procedures as Laparoscopy Bellwether procedures. Gynecologic adnexal procedures would be a composite of salpingectomy, tubal ligation, cystectomy, and oophorectomy. These Laparoscopy Bellwether procedures encompass a range of procedures from routine to emergent, are minor procedures requiring less surgeon technical expertise, yet compose the majority of laparoscopic abdominal procedures performed by programs located in LMICs [6,10,26]. Additionally, guidelines in HICs recommend laparoscopy as the first-line approach for each of these procedures [27,28,29,30,31]. Established laparoscopic programs with the capacity to consistently perform these procedures can be considered functional, with the potential to add advanced procedures to the program.

## Site Selection

Publications of underperforming or failed laparoscopy programs exist but are likely underreported [6,24]. This is a meaningful loss: resources and equipment consumed by these efforts represent great opportunity cost. It also underscores how critical site selection is for laparoscopy programs, ensuring resources are committed to locations positioned for success.

Currently, most site selection depends upon pre-existing institutional relationships [1,2,21]. This process may lack objective assessment of the recipient partner’s needs and ability to sustain a laparoscopy program, potentially missing an opportunity to build a program with greater impact. Similarly, LMIC institutions capable of benefitting from a laparoscopy program can be disadvantaged if they lack existing relationships. This does not uniformly imply that LMICs are incapable of developing laparoscopy programs without assistance, or that laparoscopy programs borne of existing institutional relationships will inevitably fail. Restricting program development with this approach, however, produces inefficiencies and suboptimal resource allocation.

Impartial criteria for appropriate site selection developed by implementing organizations and professional societies can optimize resource utilization. Important factors to consider include the presence of a functional underlying safe surgery program to ensure laparoscopic complications can be managed. Institutions that train providers who remain in-country or work with underserved or disadvantaged populations can promote access by larger populations. Additionally, organizations possessing strong research infrastructure and experience will facilitate program assessment and assist with training additional investigators [16].

## Stakeholder Inclusion

Including a comprehensive group of stakeholders increases the ability of a laparoscopy program to respond to challenges and threats; increases responsiveness and inclusion; determines priorities and standards; and improves coordination, efficiency, and resource mobilization [16,32]. Each stakeholder provides different skills, resources, and perspectives to the multidisciplinary effort (Table 1).

**Table 1:** Stakeholder Partnerships for Laparoscopic Surgery Program Development.

Stakeholder Group	Subgroups	Contribution

Operative Clinicians	General Surgeons Gynecologic Surgeons Anesthesiologists Advanced Practice Providers Nursing	Clinical training and oversight.

Peri-Operative Clinicians	Primary Care Providers Emergency Physicians	Identify patients preoperatively and assess post-operative complications.

Professional Societies		Organize site selection process, develop standards, coordinate resources, and oversight.

Research Workforce		Develop data for program assessment and site selection.

Clinical and Administrative Support Staff	Clinical Engineering Hospital Administration	Maintain equipment and provide logistical support.

Philanthropy and Global Health Funding Sources		Finance programs and oversight.

Government, Public Institutions, and Regulatory Bodies		Facilitate technological access, coordinate human and material capital.

Private Industry	Surgical and Medical Device Engineers Accelerators Device Representatives	Foster design and development of devices for low-resource settings. Maintain, repair, and replace devices.

Patients		Utilize services and provide feedback.

Surgeons, gynecologists, anesthetists, nurses, clinical engineers, and professional societies should be included in programmatic development. Anesthetists are of critical importance, as safe general and neuraxial anesthesia is fundamental to laparoscopy. A holistic approach that includes referring providers (e.g. primary care providers) and those who assess patients post-operatively (e.g. emergency medicine providers), may also improve access and outcomes.

International professional societies often have members from LMICs and affiliated local and national professional groups. Collaborations between societies can facilitate laparoscopy program development by leveraging trust, generating diverse viewpoints, and maintaining greater objectivity and impartiality in selecting sites. Such associations can improve quality by implementing standardized protocols, guidelines, and evaluation metrics. Professional organizations can combine and coordinate resources, liaising between local, national, and international actors. They can also help assess potential sites and identify local providers who possess critical knowledge of local issues. Such a partnership may be similar to the American Cancer Society’s Global Capacity Development program, which provides programmatic assessments, guides organizational development, and invests in national cancer networks [33]. Participation and close collaboration with local partners will ensure that programmatic development is done in a way that considers local needs, norms, culture, and resources.

In addition, health economists, epidemiologists, and researchers are needed to collect and analyze the data to assess program performance, identify shortcomings, evaluate new potential sites, and seek funding. Training research and development personnel in LMICs to analyze these programs from inception can increase the workforce capacity, improve local relevance of research, and, over time, ensures that the LMIC program can partner in future multinational projects [16].

Government Ministries of Health, regulatory bodies, and public-sector institutions are key to developing laparoscopy programs as they know local conditions and can coordinate larger, complex efforts. Collaborating with such institutions can guide novel technology introduction by streamlining regulatory barriers, efficiently allocating human and physical capital, and provisioning logistical and technical support. In particular, expedited device review may reduce the costs and risks of seeking regulatory approval, encouraging investment from private industry [16]. For instance, the Food and Drug Administration’s Center for Drug Evaluation and Research’s expedited review pathways speed approval of critical medicines and reduce industry costs for selected priorities; regulatory bodies in LMICs could create similar mechanisms to approve novel surgical instruments in LMICs [16].

Laparoscopists in LMICs have described numerous device workarounds, implying a market for private industry to engineer devices for reusability, durability, cost, simplified maintenance, and avoidance of consumables [1,5,6,7,19,34]. These device shortcomings prove that opportunities exist for improvement to be made which consider local contexts and capacity. Startup accelerators may facilitate the identification and enabling of early-phase innovative technologies and companies, highlight strategic barriers, and provide sustainable philanthropic investment returns that can be directed towards future endeavors [16]. Market incentive creation using push (reducing the cost of research and development) and pull (creating market demand) mechanisms may attract industry to solve specific health concerns, including laparoscopy access [16,32].

An entire laparoscopy program may be suspended when donated devices become incapacitated; thus, programs require sufficiently skilled clinical engineers or private industry representatives to maintain, repair, or replace equipment [1,9,24,35]. Clustering selected laparoscopy sites within a region could facilitate industry assigning device representatives and maintenance personnel to serve specific locations.

## Developing Stable Financing Sources

To finance laparoscopy development, interest among philanthropic bodies and global health funding sources must be promoted. Surgical capacity requires long-term investment and therefore often receives lower priority than programs promising faster results [16]. Building a laparoscopy program requires years of training, complicated logistics, and expensive equipment; the population and economic impacts may take years more to realize. It may be more tempting to greenlight a program promising faster results, such as malaria net distribution, which would be cheaper, simpler to implement, and easier to evaluate outcomes.

However, long-term programs may be more politically feasible if the economic risks to service providers and governments are mitigated; social or development impact bonds may be useful funding mechanisms to achieve this [16,36,37]. Continued disbursements may be tied to program metrics, increasing the value of initially selecting locations with program assessment capacity. One example of a development impact bond is the Mozambique Malaria Performance Bond, sponsored by Nando’s restaurant chain, which collaborated with the Mozambique Ministry of Health to finance malaria prevention [16,38]. If the goal of reducing the incidence of malaria by 30% or more after year three was met, the entire principle plus 5% interest would be repaid to Nando and other investors; if not, 50% of the principle, without interest, would be repaid [16,38]. A similarly structured program could be developed for laparoscopy.

As highlighted in the above example, domestic resource mobilization, particularly in middle-income countries, may be possible through private industry foundations operating in those countries [16,37]. For these companies vested in local population health, developing markets and maintaining healthy workforces is crucial [39]. As laparoscopy is frequently performed on working-aged individuals and results in quicker return to work, foundation investments in laparoscopy would be philanthropic and self-interested [1,19]. Often, multinational organizations can use their expertise to begin programs which later transition to another funding source. An example of this is Gavi, the Vaccine Alliance’s co-financing policy, which provides startup funding for vaccination programs but then shifts to longer-term domestically mobilized resources if the program is not anticipated to ultimately reach financial sustainability [40].

It is also essential for laparoscopy programs to assess out-of-pocket patient costs. Hidden patient expenses, including fees related to hospitalizations, peri-operative services, medications, or under-the-table provider payments may be barriers to access [14,37,41]. Studies have cited a lack of insurance coverage as creating an economic barrier to laparoscopy access [6,42]. Structural incentives – financial or otherwise – that promote or impede laparoscopy access must also be identified to ensure that patients may access laparoscopic procedures [32].

## Conclusion

Rigorous programmatic evaluation, involvement of key community, government and health care stakeholders, and development of stable financing options are required for sustainable laparoscopy programs in LMICs. To improve and standardize assessment of access to laparoscopy globally, we propose four Laparoscopic Bellwether procedures, appendectomy, cholecystectomy, gynecologic adnexal procedures and diagnostic laparoscopy. Laparoscopic surgery capacity, measured by the safe, timely, and affordable availability of these procedures is needed to ensure the most effective surgical care for all patients worldwide.

## Author Declaration Statement

The contents of this viewpoint have not been previously published, nor is it being considered for publication elsewhere.
